# Targeting immune cell trafficking and vascular endothelial cells in psoriasis

**DOI:** 10.1172/JCI169450

**Published:** 2023-05-01

**Authors:** Kelly Z. Young, Olesya Plazyo, Johann E. Gudjonsson

**Affiliations:** 1 University of Michigan Medical School and; 2 Department of Dermatology, University of Michigan, Ann Arbor, Michigan, USA.

## Abstract

The role of the vasculature in inflammatory skin disorders is an exciting area of investigation. Vascular endothelial cells (ECs) play instrumental roles in maintaining the vascular barrier and control of blood flow. Furthermore, ECs contribute to a variety of immune responses, such as targeting immune cells to specific areas of vascular damage, infection, or foreign material. However, mechanisms through which ECs participate in immune-mediated responses remain to be fully explored. In this issue of the *JCI*, Li, Shao, et al. report on vascular endothelial glycocalyx destruction and the mechanisms through which EC dysfunction contributes to the well-characterized immune-mediated features of psoriasis, a chronic inflammatory skin disease. Here, we discuss the implications of these findings and highlight some risks and benefits of existing therapies designed to target immune cell trafficking in a variety of inflammatory conditions.

## Vascular endothelial cells in psoriasis

Psoriasis is a chronic, T cell–mediated, inflammatory disease, characterized by elevated erythematous plaques, sharply demarcated from normal skin, with an overlying silvery scale (1). Although psoriasis is primarily thought to affect the skin and joints, several epidemiological studies have linked psoriasis with vascular comorbidities, supporting a potential role of vascular dysfunction in psoriasis (2). Transcriptomic profiling of vascular ECs from patients with psoriasis has revealed proinflammatory signatures, particularly those involved in inflammasome signaling, that correlate with disease severity (3). Thus, further examination of endothelial health may identify therapeutic pathways to target in patients with psoriasis.

Vascular endothelial cells (ECs) contribute to a variety of key immune responses, including but not limited to immune cell trafficking, activation, adhesion, and transmigration (4). ECs comprise a widely heterogeneous population of cells, which likely contribute to their ability to accomplish these diverse functions (4). Several recent investigations have highlighted the heterogeneity of skin ECs in morphology, localization, and function (4, 5). Li, Shao, and authors utilized a single-cell transcriptomic approach to identifying a subset of *IGFBP7^hi^* ECs from patients with psoriasis (6). Within psoriatic skin, this subset of ECs localized to papillary dermal vessels, whereas *IGFBP7^lo^* ECs localized to subpapillary dermal vessels (6).

## Endothelial glycocalyx destruction and immune cell trafficking

The endothelial glycocalyx is a dynamic, complex network of macromolecules, primarily glycoproteins and proteoglycans, that is found on the luminal surface of ECs (4). The glycocalyx forms a structural framework for binding plasma proteins and soluble glycosaminoglycans, contributing to endothelial barrier function and mechanosensation (4). Glycocalyx integrity, synthesis, and degradation can be affected by the shearing forces of turbulent blood flow and the presence of secreted factors, including angiocrine factors released by ECs (4). IGFBP7 is one such angiocrine factor that may be involved in the shedding of the endothelial glycocalyx through its known interaction with heparan sulfate, a major component of the glycocalyx (4).

**Figure 1:**
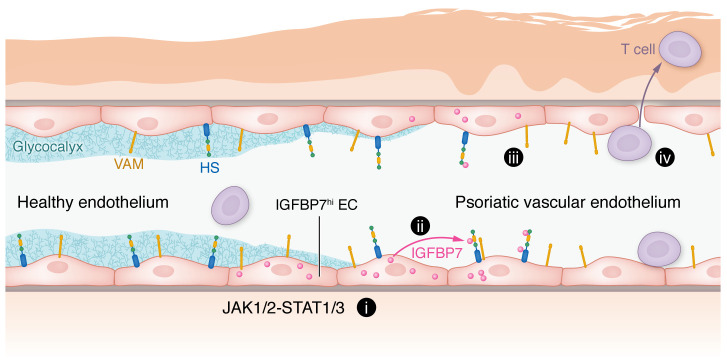
IGFBP7 in vascular ECs contributes to psoriasis. IGFBP7^hi^ vascular ECs respond to JAK1/2-STAT1/3 signaling and overexpress and secrete IGFBP7 (i). IGFBP7 then binds to components of the glycocalyx, such as heparan sulfate (HS) (ii). The endothelial glycocalyx deteriorates, increasing vascular permeability. Degradation of the endothelial glycocalyx exposes various vascular adhesion molecules (VAM) (iii) to promote T cell adhesion and transmigration (iv).

Li, Shao, and colleagues identified degradation of the endothelial glycocalyx in psoriatic skin (Figure 1), and demonstrated that IGFBP7^hi^ cells express and secrete IGFBP7 in response to psoriasis-related cytokine signaling (6). The authors further demonstrated that IGFBP7 mediated glycocalyx destruction, dampened endothelial barrier function, enhanced T cell adhesion to ECs through binding to heparan sulfate, and promoted immune cell infiltration (Figure 1). Thus, the authors provide insight into a mechanism of vascular-mediated immune cell trafficking in psoriasis. As IGFBP7 is a known angiocrine factor, the authors proposed that glycocalyx destruction in psoriasis occurs in a paracrine manner. However, since IGFBP7 does not directly enzymatically degrade the glycocalyx, additional factors likely remain to be identified in EC glycocalyx degradation. Future studies are required to further elucidate pathomechanisms of glycocalyx destruction and immune cell trafficking in psoriasis.

## Potential therapeutic strategies

Using an antibody targeting IGFBP7 in a mouse model of psoriasis, Li, Shao, and authors demonstrated that antibody-mediated blockade of IGFBP7 may be a feasible therapeutic strategy in the management of psoriasis that targets an alternative immunologic pathway: immune cell trafficking and transmigration (6). Current therapeutics in clinical use in psoriasis target several cytokines, particularly TNF or IL-12, IL-13, IL-17, and IL-23, with high efficacy and are well tolerated (7, 8). However, the data from Li, Shao, et al. and historical therapies for psoriasis provide the foundation for an alternative therapeutic approach in psoriasis.

Previous efforts targeting T cell trafficking in psoriasis included the use of efalizumab, which is a humanized monoclonal IgG1 antibody against CD11a, a subunit of lymphocyte function-associated antigen 1 (LFA-1) (9). Efalizumab inhibits binding between LFA-1 and intercellular adhesion molecule 1 (ICAM1) to affect several T cell pathways involved in psoriasis, including T cell activation, adhesion, and transendothelial trafficking (9). Several promising early clinical trials reported rapid improvement in psoriasis with efalizumab, supporting the idea that targeting T cell trafficking may be an effective strategy for psoriasis treatment (9, 10). However, efalizumab was voluntarily withdrawn from the market in 2009 due to reported incidences of progressive multifocal leukoencephalopathy (PML), a rare and fatal neurological disease caused by the reactivation of the John Cunningham (JC) virus (10).

An alternative approach to targeting immune cell trafficking for the treatment of psoriasis included the blockade of E-selectin with a humanized monoclonal antibody, CDP850 (11). E-selectin is an endothelial adhesion molecule that mediates immune cell adhesion to vascular ECs (11). E-selectin expression was found to be increased on the luminal side of vascular ECs found within psoriatic plaques, with relatively low expression found in ECs of normal skin (11). Although antibodies against E-selectin were initially found to block the recruitment of neutrophils and lymphocytes in animal models of skin inflammation, a multicenter, randomized, placebo-controlled trial in patients with psoriasis found that administration of CDP850 did not result in an improvement in PASI scores, neutrophil counts, or lymphocyte counts in the dermis (11). However, the lack of clinical difference with CDP850 in this trial may have been due to insufficient dosing or poor binding of CDP850 to human E-selectin (11).

Outside of psoriasis, targeting immune cell trafficking has also been investigated for the treatment of other inflammatory conditions, supporting the feasibility of this strategy. Natalizumab is a recombinant humanized antibody used for the management of inflammatory bowel disease and multiple sclerosis and designed to target the α_4_ chain of integrin heterodimers on leukocytes to prevent their interactions with vascular cell adhesion molecule 1 (VCAM-1), thus inhibiting leukocyte adhesion and vascular transmigration (12, 13). However, blocking α_4_β_1_ was later found to also be linked to an increased risk of PML through impaired T cell trafficking to the brain (13, 14). Thus, although targeting immune cell trafficking may provide an exciting therapeutic avenue to pursue, additional research is required for identifying ways to navigate the risks and benefits potentially associated with this therapeutic approach (10).

To circumvent the risk of PML, vedolizumab was developed for the treatment of inflammatory bowel disease (13). Vedolizumab binds to the α_4_β_7_ integrin to inhibit T cell trafficking to the intestinal epithelium (13). As α_4_β_7_ expression is largely restricted to the ECs of the gastrointestinal tract and gut-associated lymphatic tissue, the theoretical risk of PML is decreased (15). Li, Shao, et al. demonstrated that inhibition of IGFBP7 restored the endothelial glycocalyx and reduced skin inflammation in a mouse model of psoriasis, likely through its effects on T cell adhesion and trafficking (6). When determining whether IGFBP7 may provide a reasonable molecular therapeutic target in human disease, it is important to examine the systemic effects of IGFBP7 blockade to better understand the potential risks and limitations to therapeutically altering immune cell trafficking.

## Conclusions and future directions

In conclusion, Li, Shao, et al. revealed several key mechanistic features of immune cell trafficking in psoriasis, involving endothelial glycocalyx destruction, T cell adhesion, and T cell trafficking. Furthermore, the authors identified and proposed a therapeutic target, IGFBP7. The role of IGFBP7 in psoriasis is a particularly exciting discovery because IGFBP7 is thought to act on a different aspect of immune cell trafficking compared with the targets of similar existing therapies, efalizumab, natalizumab, and vedolizumab (6). At this time, several key questions remain. IGFBP7-mediated glycocalyx destruction occurs through a nonenzymatic process, suggesting the involvement of additional molecular players. Identification of these other factors would help provide further insight into the mechanism and help hone therapeutic strategies for treating psoriasis. Additionally, as psoriasis encompasses several disease stages and has a wide range of disease severity, it is important to determine whether and at what specific disease stages this type of intervention may be most effective. Finally, given the lessons from other therapeutics designed to alter immune cell trafficking in inflammatory disorders, additional studies should be pursued for understanding the potential risks of preventing glycocalyx destruction and T cell trafficking in organs outside of the skin.

## Version 1 05/01/2023

Electronic publication
